# Whole transcriptomic and proteomic analyses of an isogenic *M*. *tuberculosis* clinical strain with a naturally occurring 15 Kb genomic deletion

**DOI:** 10.1371/journal.pone.0179996

**Published:** 2017-06-26

**Authors:** Carla Duncan, Frances B. Jamieson, JoLynn Troudt, Linda Izzo, Helle Bielefeldt-Ohmann, Angelo Izzo, Carolina Mehaffy

**Affiliations:** 1 Public Health Ontario, Toronto, ON, Canada; 2 Department of Laboratory Medicine and Pathobiology, Faculty of Medicine, University of Toronto, Toronto, ON, Canada; 3 Department of Microbiology, Immunology and Pathology, Colorado State University, Fort Collins, CO, United States of America; 4 School of Veterinary Science, University of Queensland, Gatton, QLD, Australia; 5 School of Chemistry and Molecular Biosciences, University of Queensland, St Lucia, QLD, Australia; 6 Australian Infectious Diseases Research Centre, University of Queensland, St Lucia, QLD, Australia; St Petersburg Pasteur Institute, RUSSIAN FEDERATION

## Abstract

Tuberculosis remains one of the most difficult to control infectious diseases in the world. Many different factors contribute to the complexity of this disease. These include the ability of the host to control the infection which may directly relate to nutritional status, presence of co-morbidities and genetic predisposition. Pathogen factors, in particular the ability of different *Mycobacterium tuberculosis* strains to respond to the harsh environment of the host granuloma, which includes low oxygen and nutrient availability and the presence of damaging radical oxygen and nitrogen species, also play an important role in the success of different strains to cause disease. In this study we evaluated the impact of a naturally occurring 12 gene 15 Kb genomic deletion on the physiology and virulence of *M*. *tuberculosis*. The strains denominated ON-A WT (wild type) and ON-A NM (natural mutant) were isolated from a previously reported TB outbreak in an inner city under-housed population in Toronto, Canada. Here we subjected these isogenic strains to transcriptomic (via RNA-seq) and proteomic analyses and identified several gene clusters with differential expression in the natural mutant, including the DosR regulon and the molybdenum cofactor biosynthesis genes, both of which were found in lower abundance in the natural mutant. We also demonstrated lesser virulence of the natural mutant in the guinea pig animal model. Overall, our findings suggest that the ON-A natural mutant is less fit to cause disease, but nevertheless has the potential to cause extended transmission in at-risk populations.

## Introduction

*Mycobacterium tuberculosis (Mtb)*, the causal agent of tuberculosis (TB) is now the leading cause of death due to an infectious disease, surpassing HIV in 2014 [1]. Evidence of TB in humans has been found in remains dating back more than 9000 years [2] and multiple data indicate that the bacillus co-evolved with the human race and found its niche in the lung granuloma formed by the innate immune response [3–5].

This co-evolution has resulted in a complex chronic disease influenced by multiple factors including environmental, nutritional, and genetic, as well as the inherent virulence factors associated with the bacilli. Environmental and nutritional factors play an important role in the high TB burden seen in low income countries. Similarly, environmental and socio-economic factors contribute to the high risk of acquiring and developing TB that is evident in certain demographic groups from high and middle income countries. For instance, under-housed and homeless groups are known to be at high risk for TB infection and disease [6–9]. However, it is also known that certain *Mtb* strains are better fit to cause infection and the intrinsic virulence of the organism is always a determinant factor of infection and disease. Previously, we reported the microevolution of a *Mtb* strain within one high risk group, the under-housed community in Toronto, Canada [10]. This strain, designated as ON-A, acquired different single nucleotide polymorphisms (SNPs) during at least 17 year of ongoing transmission. In addition, approximately half of the ON-A isolates presented a large genomic deletion spanning 12 genes in a 15 Kb region. Deletions are generally considered deleterious events with an overall negative impact in the virulence and transmissibility of a strain, ultimately resulting in its purge from the population [11]. Contrary to this, the 15 Kb deletion in the ON-A strain seemed to have occurred during the first of two large outbreaks and was rapidly fixed in the population, causing active TB disease in about 56% of ON-A TB cases after its emergence in 2000 [10].

We hypothesize that in certain high risk populations in which factors such as nutrition, recreational drug use and the presence of co-morbidities increase the likelihood of active TB disease, *Mtb* strains that may not be as fit to cause disease, such as the ON-A natural mutant, can still adapt and even thrive resulting in extended transmission. To address this hypothesis we performed a systems biology study focusing on both proteomic and transcriptomic analyses, supplemented with data from *in vivo* infections using the guinea pig animal model of TB. The comprehensive approach allowed us to assess the physiological status and virulence of the ON-A natural mutant and ON-A wild type strains.

## Materials and methods

### Ethics statement

All experiments involving animals were approved by Colorado State University’s Institutional Animal Care and Use Committee-IACUC (protocol #13-4509A). Animals were completely sedated using ketamine and xylazine prior to being euthanized using a 1:1 Beuthanasia and saline solution injected in the animal's heart.

### *M*. *tuberculosis* strains and culture

A representative strain from the ON-A natural mutant (NM) and ON-A wild type (WT) were chosen based on the presence or absence of the 15Kb region spanning genes Rv1358 to Rv1371 [10]. WGS of these *Mtb* strains are available at the NCBI public repository with accession numbers SAMN03018400 and SAMN03018388, corresponding to sample name WT14_04 and SRA files SRS699706 for the ON-A WT and sample name NM19_08 and SRA files SRS699697 for the ON-A NM respectively.

The strains had an identical 24MIRU-VNTR (452233444212719252212423) and spoligotype (740000007760731) while RFLP analyses clustered them in 2 highly similar pseudo-clusters [10, 12]. 24MIRU and spoligotype indicates these strains are part of the Euro-American *Mtb* lineage. WGS also revealed the isogenic nature of the strains selected for this study which differ by only 5 SNPs (S1 Table) in addition to the 15Kb deletion. Both strains were pan-susceptible to all first line anti-TB drugs (Rifampicin, Isoniazid, Ethambutol and Pyrazinamide).

For virulence studies, infectivity stocks were prepared as previously described [13]. Prior to infection, one infectivity stock from each strain was titrated to determine viable organisms (CFU) by plating into 7H11/OADC plates and total organisms determined by counting individual cells in a Petroff-Hausser chamber. Known concentrations of each strain were then used to seed BACTEC MGIT tubes and placed into a MGIT^TM^ 320 (Becton Dickinson, Franklin Lakes, NJ) to assess time to positivity and determine growth rate for each strain. For proteomics studies, *Mtb* strains were cultured in large 7H11 plates for 4 weeks and then up-scaled to 500 mL of Glycerol Alanine Salt (GAS) media [14] and incubated in agitation for 2 weeks. All cultures were performed in triplicate. For transcriptomic studies, *Mtb* strains were cultured in 7H11 plates for 4 weeks and then up-scaled to 100 mL of 7H9/OADC/0.05% Tween media for two weeks. At this point, triplicate cultures at OD_600_ of 0.2 were started and incubated for 3–4 days until an OD_600_ of 0.4–0.6 was reached.

### Guinea pig infections

Guinea pig infections were performed at Colorado State University through the mutant testing program Contract # HHSN272201000009I. Infectivity stocks of each strain were shipped to Colorado State University. Outbred Hartley guinea pigs (Charles River Laboratories, MA) were infected with a low dose (10^3^ CFU) via the respiratory route by aerosolizing the bacilli in the Madison Aerosol Exposure Chamber. Thirty five animals per strain were infected and individually housed in a controlled BSL-3 environment. All of the procedures were approved by the Colorado State University Institutional Animal Care and Use Committee. At days 0, 7, 14, 21, 28, 60, and 120 post infection (d.p.i.), 5 guinea pigs per group were humanely euthanized for determination of CFUs in the lung and spleen and for cytokine analysis as described elsewhere [15]. In addition, lung pathology analysis was done as previously described [16]. When animals showed signs of disease such as weight loss, altered rate of respiration and general appearance, these animals were euthanized irrespective of their time point grouping. Survival analysis was performed using the GraphPad Prism software and differences evaluated by the Log-rank (Mantel-Cox) test. Differences in CFUs between the strains were evaluated using the Student t-test.

### Protein extraction for proteomics studies

Whole cell lysate extracts (WCL) were obtained from *Mtb* cells harvested from triplicate GAS cultures as follows. Cells were pipetted into a 50 mL Falcon tube and washed three times with PBS. After the final wash, cells were inactivated with 15 mL of chloroform. Cells were subsequently delipidated by adding 7.5 mL of methanol to make a 2:1 Chloroform:Methanol solution (v/v) and incubated overnight in agitation. Delipidation was repeated once more. After a final centrifugation at 2800 x g for 10 min the delipidated cell pellet was dried down in a nitrogen bath and WCL was prepared as described previously [17]. WCL were then subjected to three buffer exchange cycles using 10 mM ammonium bicarbonate buffer and a 3 KDa MWCO Amicon Ultra Centrifugal Filter Unit (Millipore, Darmstadt, Germany). Culture filtrate proteins (CFP) were obtained from the GAS cultures by filtering media using a STERICAP filtration unit (0.2 μM). CFP was concentrated using a 3 KDa MWCO Amicon Ultra Centrifugal Filter Unit and then subjected to three cycles of buffer exchange using 10 mM ammonium bicarbonate. Protein in WCL and CFP was quantified using the BCA Assay (Thermo Fisher Scientific, Waltham, MA) as per manufacturer’s instructions. Then, 50 μg of protein were subjected to trypsin digestion as described elsewhere [17].

### Mass spectrometry analysis

Liquid Chromatography-Tandem Mass spectrometry (LC-MS/MS) was performed at the Proteomics and Metabolomics Facility at Colorado State University. Briefly, peptides were purified and concentrated using an on-line enrichment column (Thermo Scientific 5μm, 100 μm ID x 2cm C18 column). Subsequent chromatographic separation was performed on a reverse phase nanospray column (Thermo Scientific EASYnano-LC, 3μm, 75 μm ID x 100mm C18 column) using a 90 minute linear gradient from 10%-30% buffer B (100% ACN, 0.1% formic acid) at a flow rate of 400 nL/min. Peptides were eluted directly into the mass spectrometer (Thermo Scientific Orbitrap Velos) and spectra were collected over a m/z range of 250–2000 Da using a dynamic exclusion limit of 2 MS/MS spectra of a given peptide mass for 30 s (exclusion duration of 90 s). Compound lists of the resulting spectra were generated using Xcalibur 2.2 software (Thermo Scientific, Waltham, MA) with a S/N threshold of 1.5 and 1 scan/group.

#### Mass spectrometry data analysis

Raw data were converted into mzXML format using msConvert (http://proteowizard.sourceforge.net/tools.shtml) and then searched against a *M*. *tuberculosis* database retrieved from UniProt (www.uniprot.org) with Proteome ID UP000001584 containing 3996 proteins and subsequently built to include an equal number of reverse decoys. This search was performed using the Sorcerer^TM^ software (Sage-N Research, Inc) and Sequest v1.0 (Thermo Fisher Scientific, San Jose, CA) with the following parameters: Parent Tolerance of 20 ppm, Fragment Tolerance of 1 Da, up to 4 trypsin missing sites and methionine oxidation (+16 Da) and carbamidomethylation of cysteines (+57 Da) as variable modifications. Processed files were validated in Scaffold v4.0. Peptide identifications were accepted if they could be established at greater than 95% probability by the Scaffold Local FDR algorithm. Protein identifications were accepted if they could be established at greater than 99% probability and contained at least two identified peptides. Protein probabilities were assigned by the Protein Prophet algorithm [18]. Proteins that contained similar peptides and could not be differentiated based on MS/MS analysis alone were grouped to satisfy the principles of parsimony. The Normalized Spectral Abundance Factor (NSAF) was applied to the data, directly on Scaffold and the normalized quantitative values for each protein were exported to Excel. Differences between normalized spectral counts for each strain were determined by a two-tailed Student T test. P-values of < 0.05 were considered to be significant. The mass spectrometry proteomics data have been deposited to the ProteomeXchange Consortium via the PRIDE [19] partner repository with the dataset identifier PXD005687 and 10.6019/PXD005687.

### RNA extraction for transcriptomic studies

Cells were harvested from log-phase cultures and washed three times with PBS. The cell pellet was then resuspended in 10 mL of Trizol (Thermo Fisher Scientific, Waltham, MA). PBS washes allow the removal of non-viable and dead cells so that the final extracted RNA originates only from viable cells. All samples were processed at the same time, so potential RNA degradation during the PBS washes does not limit our ability to make sample-to-sample comparison in downstream analyses. Cell pellets were vortexed and immediately frozen and kept at -80°C until further use. Immediately prior to RNA extraction, cell pellets were thawed on ice and then cells were broken by sonication on ice (50% duty cycle, 1min on, 1 min off, 6 times). Two mL of chloroform were then added to each sample and centrifuged for 30 min at 27,0000 x g, 4°C. The upper layer was transferred to a 50 mL falcon tube and mixed with 5 mL of isopropanol. RNA was precipitated overnight at -80°C. Then, samples were slightly thawed and centrifuged at 2,800 x g for 50 min at 4°C. RNA pellet was washed once with 700 μl of 80% Ethanol and finally resuspended in 200 μL of RNAse free water.

#### RNA-seq

Libraries were prepared using Illumina TruSeq stranded mRNA sample prep kit with Epicentres’ Ribo-Zero Magnetic Gold kit for ribosomal RNA depletion prior to fragmentation and priming instead of the oligo dT enrichment step. Nine libraries were run on a single lane on the Illumina HiSeq2500 using V4 Chemistry and reagents, 51 bp reads.

#### Data analysis for RNA-seq

Single end reads were aligned to *M*. *tuberculosis* H37Rv genome (NCBI accession NC_000962) using CLC Genomics Workbench 7.5, gene regions only in a strand specific manner (reads mapping to rRNA were not counted), allowing a maximum of 10 hits per read. Statistical analysis was done using the Empirical analysis of DGE tool estimating tagwise dispersions with a total filter cut off of 5 and FDR corrected p-values. Differentially expressed genes were considered significant when they presented a fold change of ≥ 1.5 or ≤ -1.5 with a p-value < 0.01 when comparing the ON-A NM to ON-A WT. These genes were then categorized according to the function as per TubercuList (http://tuberculist.epfl.ch/). Raw data has been deposited in the NCBI database with accession numbers SRX2536569 and SRX2536568 for ON-A WT; and SRX2536567, SRX2536566 and SRX2536565 for ON-A NM.

#### qRT-PCR

PCR primers were designed using Primer-BLAST (NCBI, S2 Table). The same RNA samples that were used for RNA-seq analysis were used for qRT-PCR. RNA was first treated with TURBO DNase and removal reagents and the High Capacity RNA to cDNA kit was used for cDNA synthesis (Life Technologies, Waltham, MA). RT-PCR analysis was performed with a 7900 HT Fast Real-Time PCR System (Applied Biosystems, Waltham, MA) using Power SYBR Green PCR Master Mix (Life Technologies, Waltham, MA). Two to three biological replicates were assayed in triplicate for each sample. Relative fold changes in expression level were obtained using the Pfaffl method [20] using *sigA* as a reference gene.

## Results

### Lower virulence of ON-A natural mutant in the guinea pig animal model of tuberculosis

**Fig 1:**
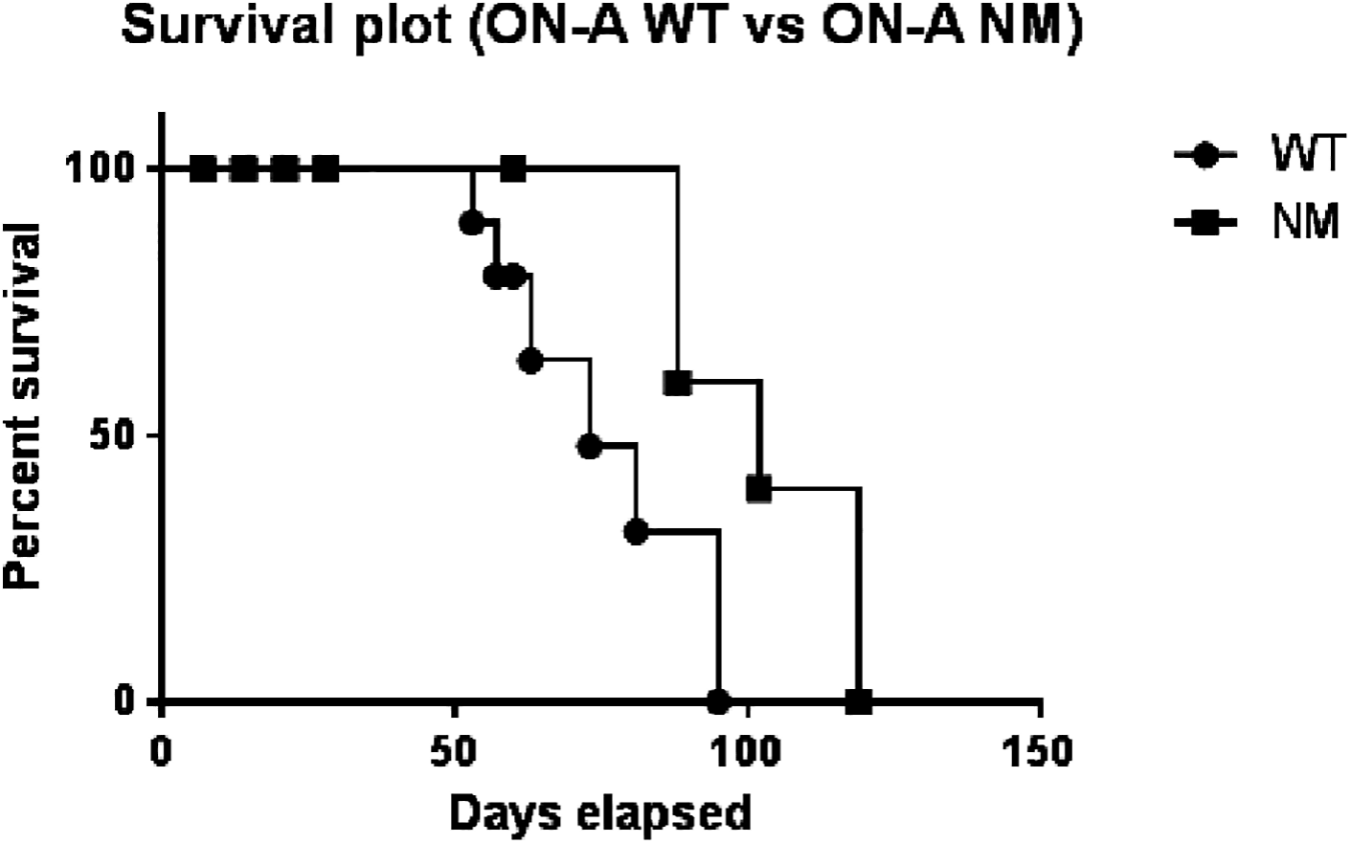
Survival analysis of guinea pigs infected with ON-A NM and ON-A WT. Thirty five guinea pigs per strain were infected with ON-A NM (Closed squares) or ON-A WT (closed circles). Log-rank test indicates the two strains differ in their ability to cause rapid disease (p-value <0.05).

Animals in both groups started to show significant signs of infection from 60 d.p.i onward, however we observed substantial differences between the two groups. For instance, all animals infected with the ON-A WT had to be euthanized before they reached the scheduled 90 d.p.i due to extent of the disease. In contrary, some animals in the ON-A NM group were well enough to continue until day 120 p.i. Given these differences, we were able to evaluate the virulence of the strains not only by histopathology analysis and bacterial burden in animals of each group, but also by performing a survival curve analysis using the Log-rank (Mantel-Cox) test. The Log-rank test indicated the two strains showed significant differences in their ability to cause rapid disease (p-value = 0.0164) with a median survival of 73 days for animals infected with ON-A WT and 102 days for the ON-A NM infected animals (Fig 1).

**Fig 2:**
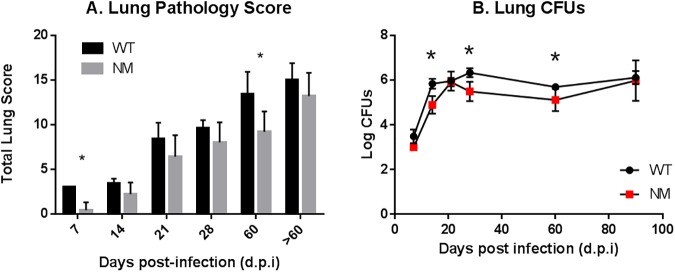
Lung pathology and CFUs of infected guinea pigs. (A) Lung pathology scores of lung of guinea pigs infected with ON-A WT (black bars) and ON-A NM (grey bars. (B) CFUs in the lung of guinea pigs infected with ON-A WT (black circles) and ON-A NM (red squares). Each point is represented by 5 animals per strain. The 90 day post infection point includes animals euthanized at days 78 (n = 2) and 108 (n = 3) for the ON-A NM group and days 63 (n = 1), 73 (n = 1), 82 (n = 1) and 96 (n = 2) for the ON-A WT group. * Statistically significant difference (p-value < 0.05).

Analysis of lung lesions also demonstrated decreased pathology in the lungs of animals infected with the ON-A NM when compared to ON-A WT at all times throughout infection with statistical significance at day 7 (p-value = 0.0079) and day 60 (p-value = 0.0359) (Fig 2). In particular, animals infected with the ON-A NM displayed less lung involvement, necrosis and fibrosis at day 60 p.i. compared to the ON-A WT infected animals (S1 Fig). Differences in lung pathology were consistent with the lower lung CFU counts observed in animals infected with the NM when compared to WT at all time points throughout infection, with the exception of day 20 and then 90 d.p.i in which a spike in NM bacterial burden was observed (Fig 2). It is important to highlight that the last time point (90 d.p.i) included animals euthanized at days 78 (n = 2) and 108 (n = 3) for the NM as opposed to animals in the WT group which had to be euthanized at earlier time points, specifically at days 63 (n = 1), 73 (n = 1), 82 (n = 1) and 96 (n = 2) due to increase in malaise and other signs and symptoms of disease. Bacteria burden in the spleen also presented a similar trend with higher numbers for the ON-A WT throughout the infection up to day 28 p.i. leveling up with the ON-A NM for day 60 onward, however these differences were not statistically significant (data not shown). Spleen pathology suggested a similar extent of disease for both strains, with slightly higher lesion type for the ON-A NM on day 28 onward although these differences were not statistically significant (data not shown). In summary, it appears the ON-A NM has the same ability as ON-A WT to disseminate in the host, however the pulmonary disease is less extensive in the ON-A NM leading to a longer survival rate in the guinea pig animal model.

Animals infected with the NM showed a decreased level of pro-inflammatory cytokines TNF-α, IFN-ɣ and IL-21 throughout the course of the infection and higher levels of IL-10. Although these observations support the lower virulence phenotype observed for the NM, there was considerable variability among individual animals and the differences were not statistically significant (data not shown).

### The 15-Kb genomic deletion results in significant changes in gene expression

Transcriptomic analysis using RNA-seq resulted in the identification and quantification of mRNA levels of 4056 transcripts, including 45 tRNAs. One repeat for WT was removed from the analysis after examining the distribution of normalization expression values (S2 Fig). After normalization and statistical analyses we identified 699 genes that presented statistically significant differences between the NM and the WT (p-value ≤ 0.01, fold change ≥1.5 or ≤ -1.5). As expected, mRNA reads mapping to all genes in the 15 Kb deletion region were significantly lower in the NM, confirming the deletion of these genes (S3 Table, S3 Fig). Fifteen targets were selected for verification by qRT-PCR. Although some of them showed different values when compared to RNA-seq (particularly *acs* and *hycQ*), all showed similar trends in expression (up or down) as observed for the RNA-seq results (S2 Table). The direct comparison of RNA-seq vs. qRT-PCR is limited by potential degradation of RNA over time as qRT-PCR was performed approximately a year later than RNA-seq, as well as primer efficiencies for each of the genes (ranging from 67–103%).

Previous WGS analysis of these strains showed they also differed by 5 SNPs [10]. ON-A WT presented 3 non-synonymous substitutions in genes Rv0026 (Asp73Tyr), Rv0570 (Ala647Glu) and Rv0938 (Asp15Gly), while ON-A WT presented 2 non-synonymous substitutions in genes Rv0061 (Arg104His) and Rv0545 (Ile101Val) (S1 Table). Although it is possible that some of the differences discussed in the following sections are related to one or more of these SNPs, we believe they are most likely due to the 15Kb deletion, a major genomic difference among the two strains.

Protein-protein network for all up or downregulated genes was performed using STRING v10 (http://version-10.string-db.org/)[21] (S4 Fig and S5 Fig). The 15Kb deletion genes were also included in each dataset in an effort to identify potential interactions. In the downregulated set, Rv1364c-Rv1366c were clustered along other regulatory proteins. Rv1364c, which encodes for the potential SigF regulatory protein also showed evidence of possible association with DevR and DevS. Although the network evidence was relative to putative homologues in other species, this is particularly interesting given the downregulation of the DevS regulon in the ON-A NM which will be discussed in the sections below. In the network of upregulated genes, Rv1364 showed links to TcrY, the histidine kinase of two component system (TcrY/TcrX). This two component system is involved in iron acquisition but has not been fully characterized [22, 23]. Protein-protein networks also identified several protein clusters (S4 Fig and S5 Fig), some of which will be described in the following sections.

Proteomic analysis resulted in the identification and quantification of 1066 proteins in the WCL of which 107 were statistically different between the two strains (S4 Table). Three hundred and seven proteins were identified in the CFP of which 21 showed significant differences among the strains (S4 Table). Replicate variability was larger in the proteomics dataset which, in addition to intrinsic differences in proteomics and transcriptomics methodology and data analysis as well as biological factors such as mRNA and protein stability, may account for the smaller set of proteins with statistical significance when compared to the RNA-seq results and for the inconsistencies in protein vs mRNA abundance observed in nearly 50% of all significantly different proteins between ON-WT and ON-A NM (S4 Table). This is in accordance with several studies that have highlighted the difficulties in attaining the same level of coverage in proteomics vs. transcriptomics studies, including a poor correlation between the two due to post-transcriptional and post-translational modifications, translation rates, protease activity and subcellular localization [24–27]. In particular for *Mtb*, differences between protein level and gene expression have been attributed to be due to the dynamic nature of the pathogen [28], as well as a generally long half-life mRNA [29] and high protein turnover [30]. In addition, gene specific differences in both mRNA and protein stability could contribute to the differences observed between transcriptomic and proteomics results. For instance, the hypoxia hallmark, HspX, has been shown to have inversely related mRNA and protein abundance during both log an stationary phases [31]. The catalase-peroxidase KatG has been shown to have a higher than average mRNA stability due to a post-transcriptional polypurine tail as well as translation initiation [32]. In our data, both KatG and HspX showed significant differences at the mRNA level but not at the protein level, suggesting both mRNA stability as well as protein turnover could be playing a role for some of the differences observed between transcriptomics and proteomics results between the ON-A NM and WT.

#### ON-A NM shows a shift in expression of respiration and energy production genes

Several genes coding for the cytochrome P450 system (i.e. *cyp132*, *cyp135A1*, *cyp136*, and *cyp144*) were downregulated in the NM while the *cydABCD* operon (*Rv1620c-Rv1623c*) which is part of the cytochrome bd complex was upregulated in the NM (S3 Table). The fumarate reductase operon *frdABCD* (*Rv1552-Rv1555*) was downregulated in the NM while the formate hydrogenlyase genes *hycDPQE* (*Rv0084-Rv0087*) and the pyruvate dehydrogenase complex *pdhABC*, also annotated as branched-chain keto acid dehydrogenase (*bkdABC*) [33], were upregulated in the NM (S3 Table).

#### Gene expression and protein abundance related to amino acid biosynthesis is altered in ON-A NM

Proteins involved in the synthesis of the amino acid arginine (CarA, CarB, ArgB, ArgC, and ArgG) had a lower abundance in the NM (S4 Table). CarAB (carbamoyl phosphate synthetases units A and B) catalyze the first step in pyrimidine and arginine biosynthesis by catalyzing the conversion of glutamine to carbamoyl phosphate while ArgB and ArgC are part of a 5 enzyme pathway that uses glutamate to produce ornithine. Both carbomoyl phosphate and ornithine then enter the urea cycle in which ArgG in conjunction with ArgF and ArgH activities result in arginine production. Of interest, the arginine repressor *argR* was found over-expressed in the ON-A NM while the tRNA-arg (Rvnt43) was found in lower abundance, all of these indicating a potential downregulation of the production of arginine in this strain. In contrast, the *thrACB* operon which is involved in the biosynthesis of several amino acids such as threonine, isoleucine and methionine were upregulated in the NM. Accordingly, two of the three tRNA carrying methionine were also found to be over-expressed in the NM (S3 Table).

#### Downregulation of molybdenum cofactor (MoCo) biosynthesis genes in the NM

**Fig 3:**
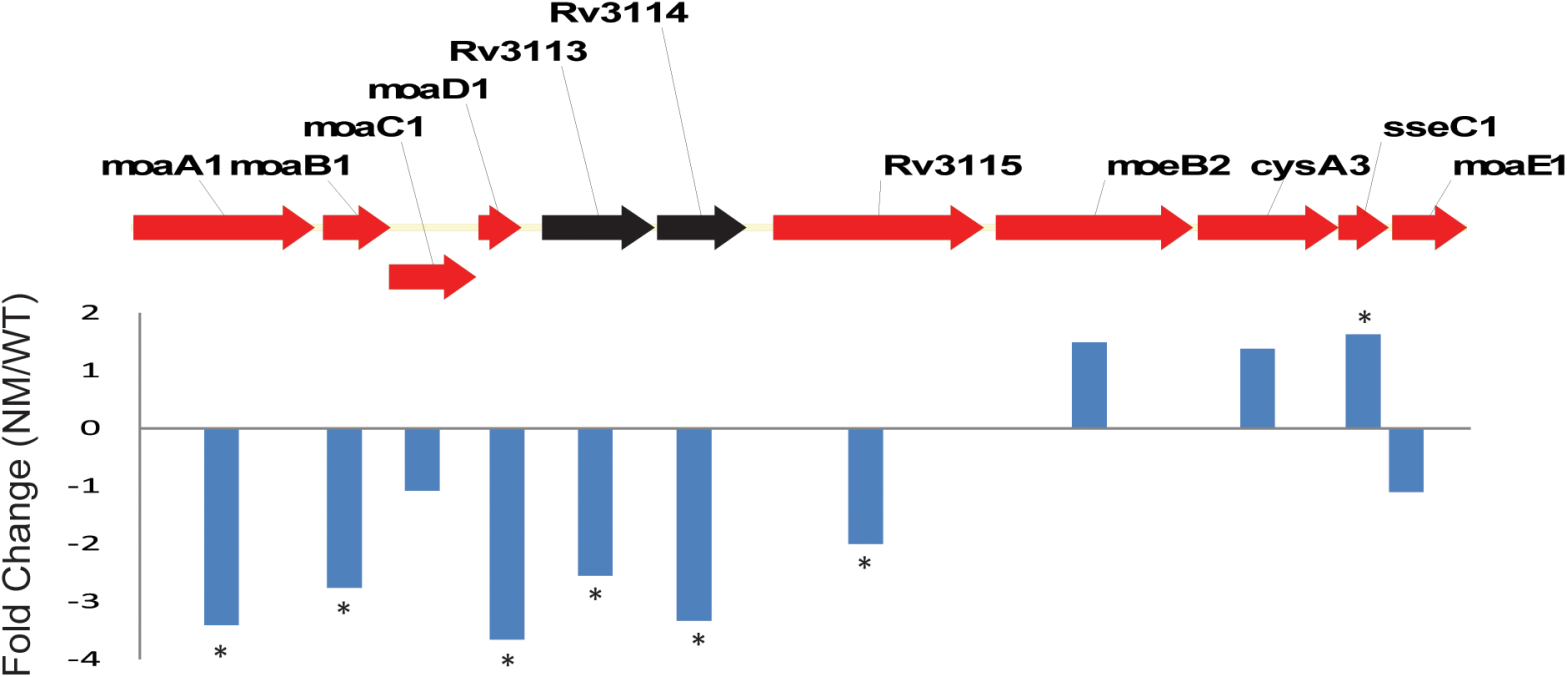
Differentially expressed genes in the molybdenum cofactor biosynthesis loci 1. Organization of genes involved in MoCo biosynthesis in red. Bars represent gene expression fold change between ON-A WT in relation to ON-A NM. *Genes with statistically significant values (p-value <0.01).

Perhaps one of the most striking differences between the two ON-A strains was the significant downregulation of MoCo biosynthetic genes in the ON-A NM. (Fig 3 and S6 Fig). In *Mtb*, MoCo genes are organized into three different loci [34]. Significantly downregulated genes were located in locus 1 (*moaA1*, *moaB1* and *moaD1*) and locus 3 (*moaC3* and *moaX)* (Fig 3 and S6 Fig). In addition to the MoCo biosynthesis genes, we also identified Rv3124 (*moaR1*), the positive regulator for MoCo biosynthesis [35], as downregulated in the NM. In contrast, *modB* and *modA* which encode the subunit B of the molybdate import system were upregulated (fold change 1.95 and 1.35 respectively), suggesting the ON-A NM is compensating for a potential decrease in intracellular MoCo. The downregulated MoCo genes, with the exception of *moaC2*, are uniquely conserved in the *M*. *tuberculosis* complex and absent in other non-pathogenic mycobacteria species [36] suggesting these are important for virulence. These results indicate that biosynthesis of MoCo in general is decreased in the natural mutant possibly as a consequence of the 15 Kb genomic deletion.

#### ON-A NM presents differential expression of genes involved in lipid metabolism

Fifty-four genes involved in lipid metabolism showed significant differences in their expression between the NM and WT. Of these, three clusters of genes are worth mentioning. First, several genes involved in the biosynthesis of mycolic acid (i.e. *accD6*, *cmaA2*, *fabD*, *fabD32*, *fas*, *kasA*, *kasB*, *acpM*) [37] were downregulated in the NM (S3 Table, S4 Fig). Second, genes involved in the phthiocerol dimycocerosates (PDIM) pathway, *ppsABCDE* as well as the putative PDIM transporter *drrABC* and *lppX* [38] were all upregulated in the mutant (S3 Table, S5 Fig). Higher abundances of Mas (Rv2940), an enzyme involved in the final steps of PDIM biosynthesis, as well as overabundance of LppX were also observed by proteomics of the whole cell lysate, supporting the overall overexpression of the PDIM biosynthetic pathway in ON-A NM (S3 Table and S4 Table). Finally, genes involved in the biosynthesis and translocation of 2,3-diacyltrehaloses (DAT) were also upregulated in the mutant (*pks3*, *pks4*, *papA3*, *fadD21*, *mmpL10* and Rv1184c) [39] (S3 Table).

#### ON-A NM has decreased expression of the hypoxia regulon DevS-DevR

DevS-DevR is one of the most studied two component systems in *M*. *tuberculosis* due to its central role in the response to hypoxia and nitric oxide (NO) by the bacilli [40–44]. Here we identified both *devS* and *devR* as significantly downregulated in ON-A NM. Albeit not statistically significant, proteomics data also supported the lower abundance of both DevR (p-value 0.057) and DevS (p-value 0.063). Given the downregulation of the *DosR* operon (*devR-devS*) which has been extensively shown to regulate a set of 48 genes under hypoxia conditions, we proceeded to interrogate our set of differentially expressed genes for those known to be part of this regulon. Twenty genes, including the hypoxia hallmark *hspX* were identified as differentially expressed in the mutant (Table 1). With the exception of *Rv0081-Rv0083*, all genes were downregulated in the mutant. The *Rv0081-Rv0083* operon is regulated not only by DevR but also by the regulator MprA and *Rv0081* itself [45]. *Rv0081* is a negative self-regulatory gene and together with DevR mediate the initial response to low oxygen levels followed by the enduring hypoxic response [46]. *Rv0081* has been recently shown to have a much broader regulatory function, showing a distinct expression pattern during hypoxia and re-aeration when compared to DevR [47]. This complex regulation of *Rv0081-Rv0083* may explain their upregulation in the ON-A NM as opposed to the remaining 17 DosR-regulated genes identified as downregulated in this strain.

**Table 1:** Differentially expressed genes from the DevR regulon.

Gene name	Rv Number	Function	Normalized Fold Change
Rv3126c	Rv3126c	hypothetical protein	-2.75
narU	Rv0267	probable integral membrane nitrite extrusion protein naru (nitrite facilitator)	-2.39
Rv3128c	Rv3128c	conserved hypothetical protein	-2.30
Rv1813c	Rv1813c	conserved hypothetical protein	-2.18
Rv1733c	Rv1733c	probable conserved transmembrane protein	-2.17
hspX	Rv2031c	heat shock protein hspx (alpha-crystallin homolog) (14 kda antigen) (hsp16.3)	-2.15
Rv2005c	Rv2005c	conserved hypothetical protein	-2.11
fdxA	Rv2007c	probable ferredoxin fdxa	-1.90
Rv3127	Rv3127	conserved hypothetical protein	-1.87
devS	Rv3132c	two component sensor histidine kinase devs	-1.83
devR	Rv3133c	two component transcriptional regulatory protein devr (probably luxr/uhpa-family)	-1.82
acg	Rv2032	conserved hypothetical protein acg	-1.80
Rv1738	Rv1738	conserved hypothetical protein	-1.79
hrp1	Rv2626c	hypoxic response protein 1 hrp1	-1.75
ctpF	Rv1997	probable metal cation transporter p-type atpase a ctpf	-1.71
otsB1	Rv2006	probable trehalose-6-phosphate phosphatase otsb1 (trehalose-phosphatase) (tpp)	-1.70
Rv3134c	Rv3134c	conserved hypothetical protein	-1.68
Rv0082	Rv0082	probable oxidoreductase	1.54
Rv0081	Rv0081	probable transcriptional regulatory protein	1.87
Rv0083	Rv0083	probable oxidoreductase	1.88

## Discussion

The *Mtb* ON-A strain has circulated in the under-housed population of Toronto, Ontario, Canada since at least 1997, causing two different TB outbreaks. In a previous study by our group we identified the emergence of a 15 Kb deletion during the first TB outbreak in this population in 2001. This deletion did not cause an apparent reduction in transmission, however, given the complexity of the under-housed population which often present with comorbidities and other factors that put them at a higher risk of developing active TB disease, it is difficult to determine if this mutation had an important effect on the physiology of the bacilli, and subsequent pathophysiology within the host.

The 15 Kb deletion is comprised of 12 genes (*Rv1358 –Rv1371*). Two of them are annotated as transcriptional regulators (*Rv1358-Rv1359*) and another two are annotated as regulatory proteins, particularly involved in SigF activity (*Rv1364c-rsfA*). In addition, *Rv1366* (function currently unknown) has a functional domain associated with the RelA-SpoT superfamily which is involved in the synthesis of ppGpp, a secondary messenger molecule that is also associated with gene regulation under certain stresses [48, 49] and is required for full virulence of *Mtb* [50, 51].

Given the function of these genes we hypothesized that the 15Kb deletion may have an impact on the physiology of the organism, particularly in gene expression and potentially in the ability of the bacterium to thrive during in vivo infection in immunocompetent individuals. In order to gain insights into the physiological consequences of this genomic deletion, we infected guinea pigs with either the ON-A WT or ON-A NM strains and assessed their virulence in this model. Our findings indicate that ON-A NM had reduced virulence in the guinea pig model, indicated by a lower bacterial burden in the lungs throughout the infection, less severe pathology, and a lower mortality rate as compared to animals infected with the WT strain.

*Mtb* is exposed to an array of host induced stresses which include, but are not limited to, nitric oxide (NO), low pH, and low oxygen availability. The majority of compensating mechanisms of *Mtb* to allow it to not only survive but thrive in this environment are tightly regulated by the expression of regulons and other clusters of genes. It is possible that the deletion of all or some of the 12 genes in the ON-A NM may impact the ability of the bacteria to respond to these stresses. Our transcriptomics and proteomics analyses appear to corroborate this idea as several of the regulons and other gene clusters known to be associated with response to NO and low oxygen levels, essential for adequate intracellular survival, were found differentially regulated as compared to the WT. Perhaps the most striking finding was the overall downregulation of the DosR regulon, including the two-component regulators *devR-devS*. The DosR regulon is a well-established virulence factor associated with dormancy survival under NO and low oxygen levels [40, 41, 43, 44, 47, 52, 53]. DevR is a transcriptional regulator activated by the histidine kinases DevS or DosT (Rv2027c). DevS is inactive in the presence of O_2_, but when O_2_ levels decrease, DevS undergoes auto-phosphorylation and transfers its phosphate group to the response regulator DevR which results in upregulation of almost 50 genes. The importance of this system as a virulence factor has been highlighted by several studies in animal models [42, 54–57] where the activation of the DosR dormancy regulon is required to successfully establish infection. In addition, the highly virulent W-Beijing strain appears to have the DosR regulon constitutively overexpressed [58]. The downregulation of the DosR suggest the ON-A NM may be better suited for growth under conditions that are not oxygen limited and thus ON-A NM may have less adaptation capacity during intracellular infection. Other findings, however, indicate that ON-A NM may have developed adaptation/compensatory mechanisms such as the upregulation of the cytochrome bd oxidase, which has greater affinity for oxygen and thus is expected to be highly active during low oxygen tension [59, 60].

In addition to the DosR regulon and mycolic acid biosynthesis genes, which were downregulated in the ON-A NM, the next largest cluster of genes differentially expressed in the ON-A NM corresponded to MoCo biosynthetic genes, which were also downregulated in the mutant. MoCo is utilized by molybdenum enzymes to catalyze redox reactions in carbon, sulfur and nitrogen metabolism [36]. Genes involved in the MoCo biosynthesis pathway are expanded in *Mtb* with multiple homologues for some of the genes [36, 61]. However, despite this genetic redundancy, high throughput screening of mutants indicates that some of these genes are individually required for successful inhibition of phagosome maturation and thus, may be important for full virulence of the bacilli [62, 63]. MoCo biosynthesis is linked to cellular homeostasis of metals such as copper and iron and are also associated with cysteine and sulfur metabolism through the second step of MoCo biosynthesis which is characterized by the sulfur transfer to the molybdopterin synthases MoaD1 and MoaD2 [36, 64]. Interestingly, the sulfotransferase CysA2 was found in higher abundance in the WCL of the mutant and coding genes for CysA1 and CysH, two enzymes involved in active import of sulfate and thiosulfate, were also found upregulated in the ON-A NM, possibly as a compensating mechanism for low MoCo production.

It is possible that the deletion of genes associated with the alternative sigma factor F (SigF), such as *Rv1358* which is regulated by SigF, *Rv1364* (Potential *sigF* regulatory protein) and the anti-anti-sigma factor F, *rsfA* (*Rv1365*), all part of the 15 Kb deletion, may be directly associated with all or some of the transcriptomic and proteomic differences identified in this study. Little is known about SigF function. It appears to play a role in gene regulation during early and late stationary growth phases, as evidenced by studies of a SigF deletion mutant in which 187 and 277 genes were downregulated in each of these phases, while only 38 genes were downregulated during exponential growth [65]. Our proteomic and RNA-seq analyses were all performed using cells harvested at mid-log phase and thus we may have missed additional differences associated specifically with SigF. However, we identified 11 of the 38 SigF regulated genes as either up or downregulated in the NM. In particular, *fabD*, *acpP* and *kasA*, genes involved in lipid metabolism were found downregulated in the ON-A NM and supports previous observations on *Mtb* SigF and its potential implication in regulating the expression of genes involved in cell wall structure, lipids and polysaccharides.

To date, no particular environmental stresses have been associated with SigF activity [66] and a *sigF* deletion does not impact cell growth during in vitro culture. However, a *sigF* mutant was shown to be severely attenuated during in vivo murine infection [65]. We could hypothesize that the deletion of the anti-anti-SigF (*rsfA*) may lead to a reduction in SigF activity due to dysregulation of the anti-SigF factor. However, RsfA is only one of two antagonistic anti-SigF factors and seems to be active only under reducing conditions [67] which were not tested in this study.

In summary, our findings indicate the ON-A 15Kb genomic deletion leads to significant physiological consequences for the organism, highlighted by evidence of lower virulence in the guinea pig infection model. This is postulated to be due to the downregulation of hypoxia-induced genes and molybdenum cofactors as well as differential regulation of amino acid, lipid biosynthesis and genes involved in energy production. However, some of our findings, such as the upregulation of genes involved in PDIM and DAT biosynthesis in the ON-A NM, both of which have been implicated in virulence [68–71] may indicate compensatory mechanisms this strain uses to persist in the host, albeit causing a lesser degree of pathology. Additional studies evaluating the gene expression of ON-A NM and ON-A WT at the early phases of acute *in vivo* infection, as well as studies evaluating gene expression and protein abundance as the bacteria undergoes a shift in oxygen supply are needed to better understand the potential role of a low DosR regulon expression in the decreased pathology and CFUs observed at the early time points in the guinea pig group infected with ON-A NM. Metabolomics analysis of the strains may also provide important information to support the differential production (both up and down) of several lipid categories and other small metabolites.

Similarly, additional studies of individual deletion mutants of the 15 Kb genomic region (*Rv1358- Rv1371*) are needed in order to pinpoint the specific gene or genes contributing to the lower virulence of ON-A NM. Particularly, studies of an *rsfA* KO mutant would provide important information with regard to the regulation of *sigF* under different environmental conditions.

## Supporting information

S1 Table List of SNPs of ON-A WT and ON-A NM from WGS data [10]. (DOCX)

S2 Table qRT-PCR primers and validation of RNA-seq results. (DOCX)

S3 Table Expression data and functional categories for genes with fold-change ≥1.5 or ≤-1.5 and FDR corrected p-value <0.01 in ON-A NM relative to ON-A WT. CH–conserved hypothetical, CWCP–cell wall and cell process, IMR–intermediary metabolism and respiration, IP–information pathways, ISP–insertion sequences and phage, LM–lipid metabolism, PPE–PE/PPE gene family, RP–Regulatory Protein, UK–Unknown, VDA–virulence detoxification and adaptation.
(XLSX)

S4 Table Proteomics data: Proteins identified in the CFP (secreted fraction) or WCL (whole cell lysate) as having significantly differential abundances (p-value < 0.05) between ON-A WT and ON-A NM. CH–conserved hypothetical, CWCP–cell wall and cell process, IMR–intermediary metabolism and respiration, IP–information pathways, ISP–insertion sequences and phage, LM–lipid metabolism, PPE–PE/PPE gene family, RP–Regulatory Protein, UK–Unknown, VDA–virulence detoxification and adaptation.
(XLSX)

S1 Fig **Lung Histology Photomicrographs (20X and 40X magnification) of one guinea pig representing** A. ON-WT and B. ON-A NM at day 60 p.i.
(DOCX)

S2 Fig Boxplot showing distribution of RNA seq normalized expression values for ON-A WT and NM replicates. (PDF)

S3 Fig RNA seq reads mapping to NC_000962.3 in the 15-Kb genomic region deleted in NM strain. (PDF)

S4 Fig Predicted interaction network of genes found downregulated in the ON-A NM. (PDF)

S5 Fig Predicted interaction network of genes found upregulated in the ON-A NM. (PDF)

S6 Fig Gene organization and differential expression molybdenum cofactor biosynthesis genes loci 2 and 3. Genes involved in MoCo biosynthesis are in red. Bars represent gene expression fold change between ON-A WT in relation to ON-A NM. *Genes with statistically significant values (p-value <0.01).
(DOCX)
