# Supplementary material for: Whole transcriptomic and proteomic analyses of an isogenic M. tuberculosis clinical strain with a naturally occurring 15 Kb genomic deletion
Source: PLoS One. 2017 Jun 26;12(6):e0179996. doi: 10.1371/journal.pone.0179996 (PMC5484546; doi:10.1371/journal.pone.0179996)
Supplement: S1 Table — (DOCX) [file pone.0179996.s001.docx]

**S1 Table. List of SNPs of ON-A WT and ON-A NM from WGS data [10]**

For ON-A NM two SNPs (shaded in blue) were identified by WGS with respect to ON-A WT. For ON-A WT three SNPs (shaded in orange) were identified by WGS with respect to ON-NM. H37Rv is shown as reference.

|  | **Nucleotide position in H37Rv genome (Gene)** | | | | |
| --- | --- | --- | --- | --- | --- |
| **Strain** | 29938  (Rv0026) | 65301  (Rv0061) | 637163  (Rv0545, pitA, inorganic phosphate transporter) | 663234  (Rv0570, nrdZ, DNA replication) | 1046179  (Rv0938, ligD, DNA repair) |
| ON-A NM | **G** | **A (Arg 104His)** | **C (Ile101 Val)** | **C** | **A** |
| ON-A WT | **T (Asp73Tyr)** | **G** | **T** | **A (Ala647Glu)** | **G (Asp15Gly)** |
| H37Rv | **G** | **G** | **T** | **C** | **A** |
