# Supplementary material for: Whole transcriptomic and proteomic analyses of an isogenic M. tuberculosis clinical strain with a naturally occurring 15 Kb genomic deletion
Source: PLoS One. 2017 Jun 26;12(6):e0179996. doi: 10.1371/journal.pone.0179996 (PMC5484546; doi:10.1371/journal.pone.0179996)
Supplement: S2 Table — (DOCX) [file pone.0179996.s002.docx]

**S2 Table. qRT-PCR validation of RNA-seq result**.

Fold change of ON-A NM in relation to ON-A WT; a – Empirical analysis of DGE test, b- relative fold change determined using the Pfaff method with *sigA* as a normalizer.

|  | **Fold Change** | |  |  |  |
| --- | --- | --- | --- | --- | --- |
| **target** | **RNA-seq^a^** | **qRT-PCR^b^** | **Primer 1 forward** | **Primer 2 reverse** | PCR efficiency |
| *acs* | 7.02 | 1.16 | GTAGCGGCAAAATCATGCGT | GAATACGGTGGGATCGAGCA | 81.1 % |
| *frdA* | -4.35 | -3.34 | GACGACGAGCATTTCTTGGC | CCAGCGAGTGATAGTGACCG | 94.5 % |
| *furA* | 2.29 | 2.26 | TAACGGCTTCCTGTTGGACG | GATGTGATCGCGAAGTGTCG | 95.5 % |
| *hycD* | 1.93 | 1.40 | CAAACTGCGATTGTTCCGGG | CCCCACCGTGAAGAAGTTGG | 67.3 % |
| *hycE* | 1.85 | 1.44 | GACAGTCTGCGTGTCGTGTA | GGAAAGGACAGGTACGCCAA | 87.3 % |
| *hycQ* | 2.01 | 1.19 | CAACATCGGCGTCATTTGGG | CCACGTATTTCCAGGTCGCT | 84.8 % |
| *iniB* | -5.35 | -4.63 | CGAGCCATTCGGTGTTTGAC | GGTAGTCCGAAAAGGCCGTA | 85.2 % |
| *katG* | 3.15 | 3.52 | CAGCAGATGACGGGACCTAC | GTCCACTTCACCTTGCCACT | 99.8 % |
| *moaA1* | -3.41 | -1.99 | ACGTATTGAGCCTTTGCCCA | TGTCGACGCGATAATTCCGA | 94.7 % |
| *moaB1* | -2.76 | -3.07 | TACTGGACAAGCGTTCGGTG | CGATGTCGGTCAGATAGCCC | 91.1 % |
| *moaD1* | -3.66 | -1.17 | CCTCGGGAGGAAGTAGAGGT | ATTGGTTGACCGCCATCTGT | 101.6 % |
| *moaX* | -2.64 | -1.48 | GCCACTTGCTGATTTTCGCA | TGATGCTGGCGCTATGGATT | 98.0 % |
| *moeW* | -2.59 | -2.04 | CAACCGAACAACAACGGCTT | CGACCATGTCAATTCCCCCA | 102.6 % |
| *pks4* | 2.49 | 2.71 | CCGCTGTCCGACTATGGTTT | CCGTGGTGATCTTTGTGGGA | 94.5 % |
| *sigA* | -1.07 | - | GGTGCTGGACACGCTCTC | GTAGACCTGGCCGATCTCGT | 89.8 % |
| *sigF* | 1.03 | 1.04 | TCGATGACCCAAACGCAGAT | GTAGCCGTGCCAATGACTTG | 92.5 % |
