# Supplementary material for: Whole transcriptomic and proteomic analyses of an isogenic M. tuberculosis clinical strain with a naturally occurring 15 Kb genomic deletion
Source: PLoS One. 2017 Jun 26;12(6):e0179996. doi: 10.1371/journal.pone.0179996 (PMC5484546; doi:10.1371/journal.pone.0179996)
Supplement: S1 Fig — Lung Histology Photomicrographs (20X and 40X magnification) of one guinea pig representing A. ON-WT and B. ON-A NM at day 60 p.i. (DOCX) [file pone.0179996.s005.docx]

**S1 Fig**

| 1. **ON-A WT** | |
| --- | --- |
|  |  |
| 1. **ON-A NM** | |
|  |  |
