# Supplementary figures and images for: Whole transcriptomic and proteomic analyses of an isogenic M. tuberculosis clinical strain with a naturally occurring 15 Kb genomic deletion
Source: PLoS One. 2017 Jun 26;12(6):e0179996. doi: 10.1371/journal.pone.0179996 (PMC5484546; doi:10.1371/journal.pone.0179996)

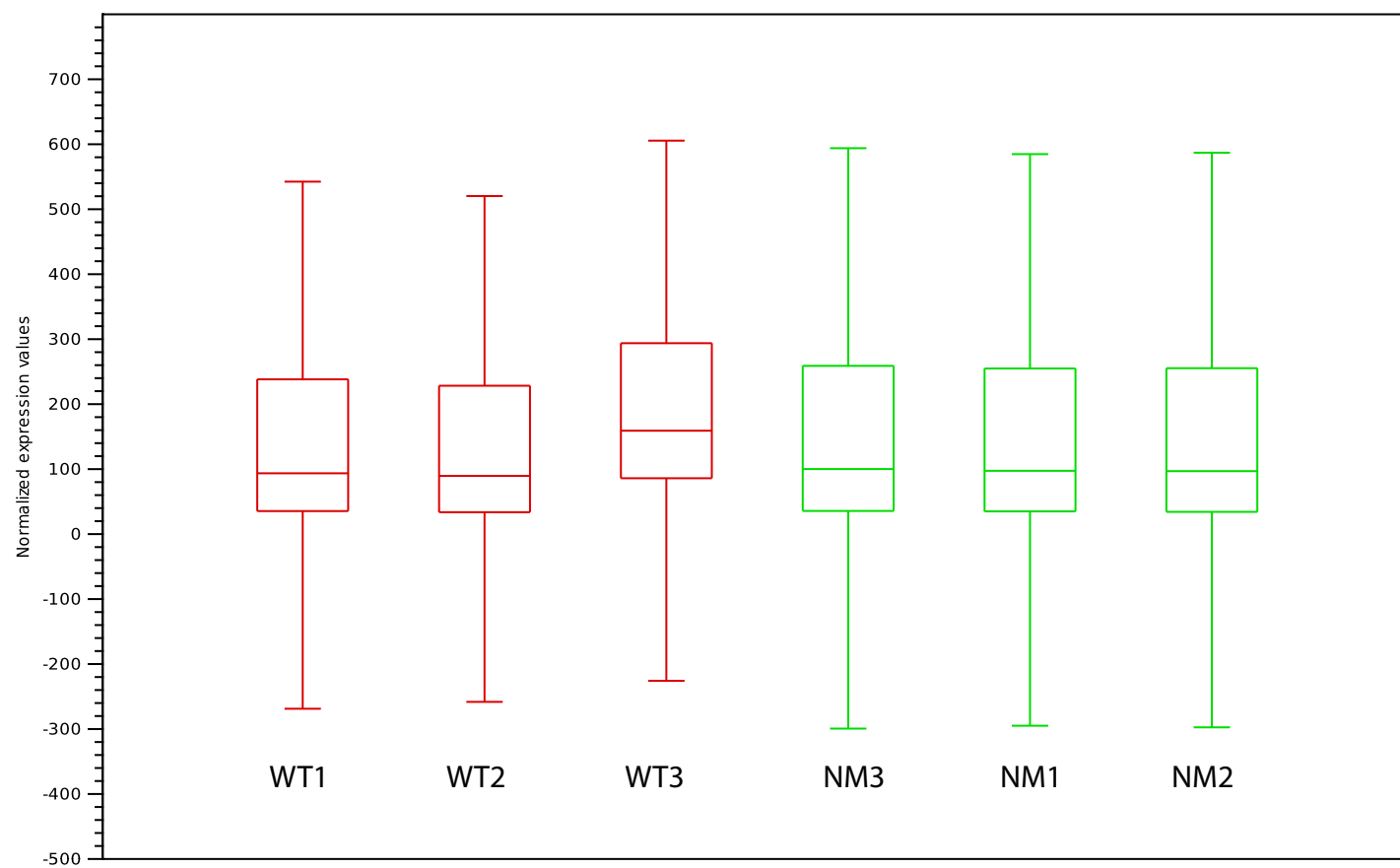

Supplement: S2 Fig — (PDF) [file pone.0179996.s006.pdf]

**S6 Fig**


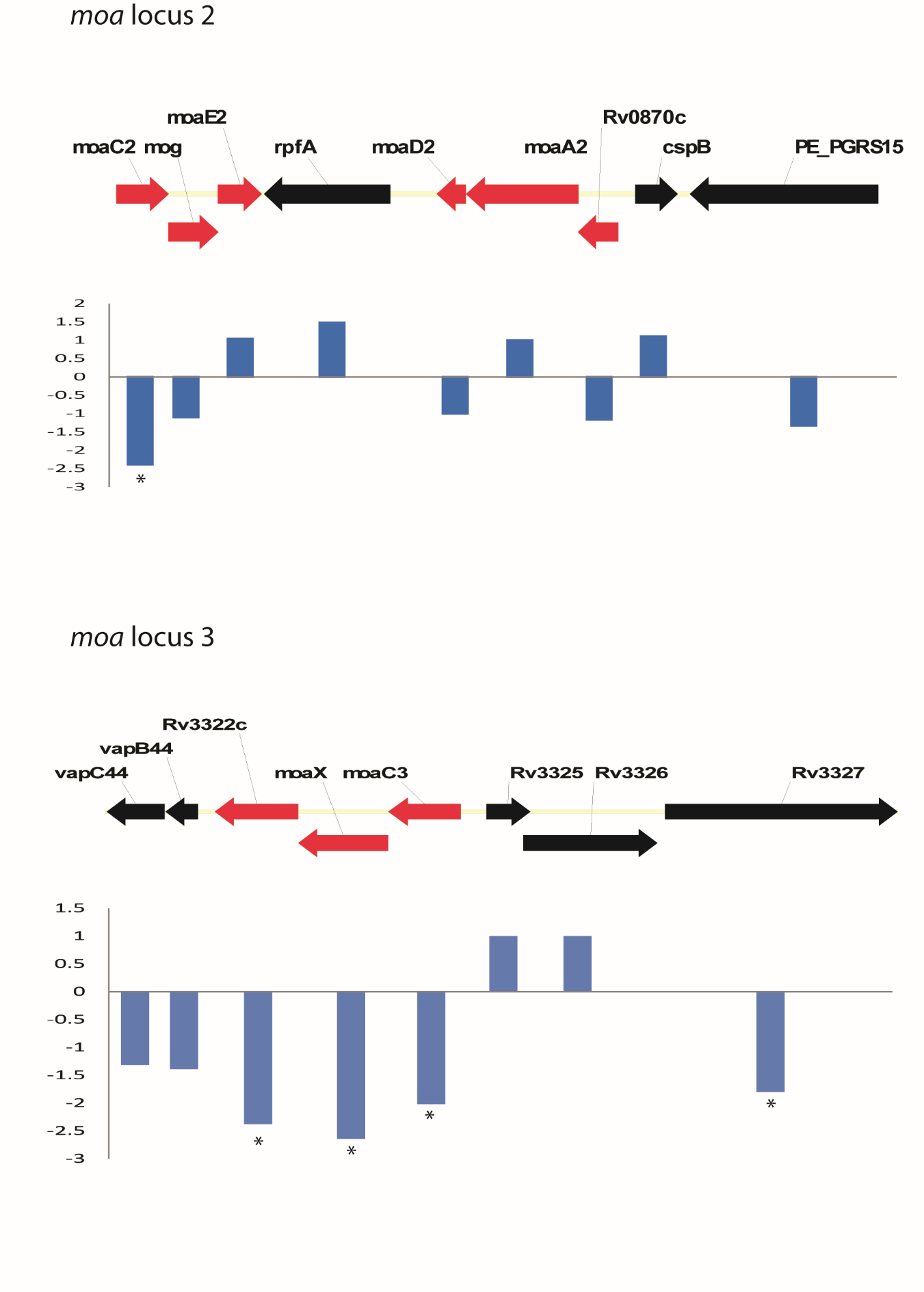

Supplement: S6 Fig — Genes involved in MoCo biosynthesis are in red. Bars represent gene expression fold change between ON-A WT in relation to ON-A NM. *Genes with statistically significant values (p-value <0.01). (DOCX) [file pone.0179996.s010.docx]
