# Supplementary material for: Whole transcriptomic and proteomic analyses of an isogenic M. tuberculosis clinical strain with a naturally occurring 15 Kb genomic deletion
Source: PLoS One. 2017 Jun 26;12(6):e0179996. doi: 10.1371/journal.pone.0179996 (PMC5484546; doi:10.1371/journal.pone.0179996)
Supplement: S3 Fig — (PDF) [file pone.0179996.s007.pdf]

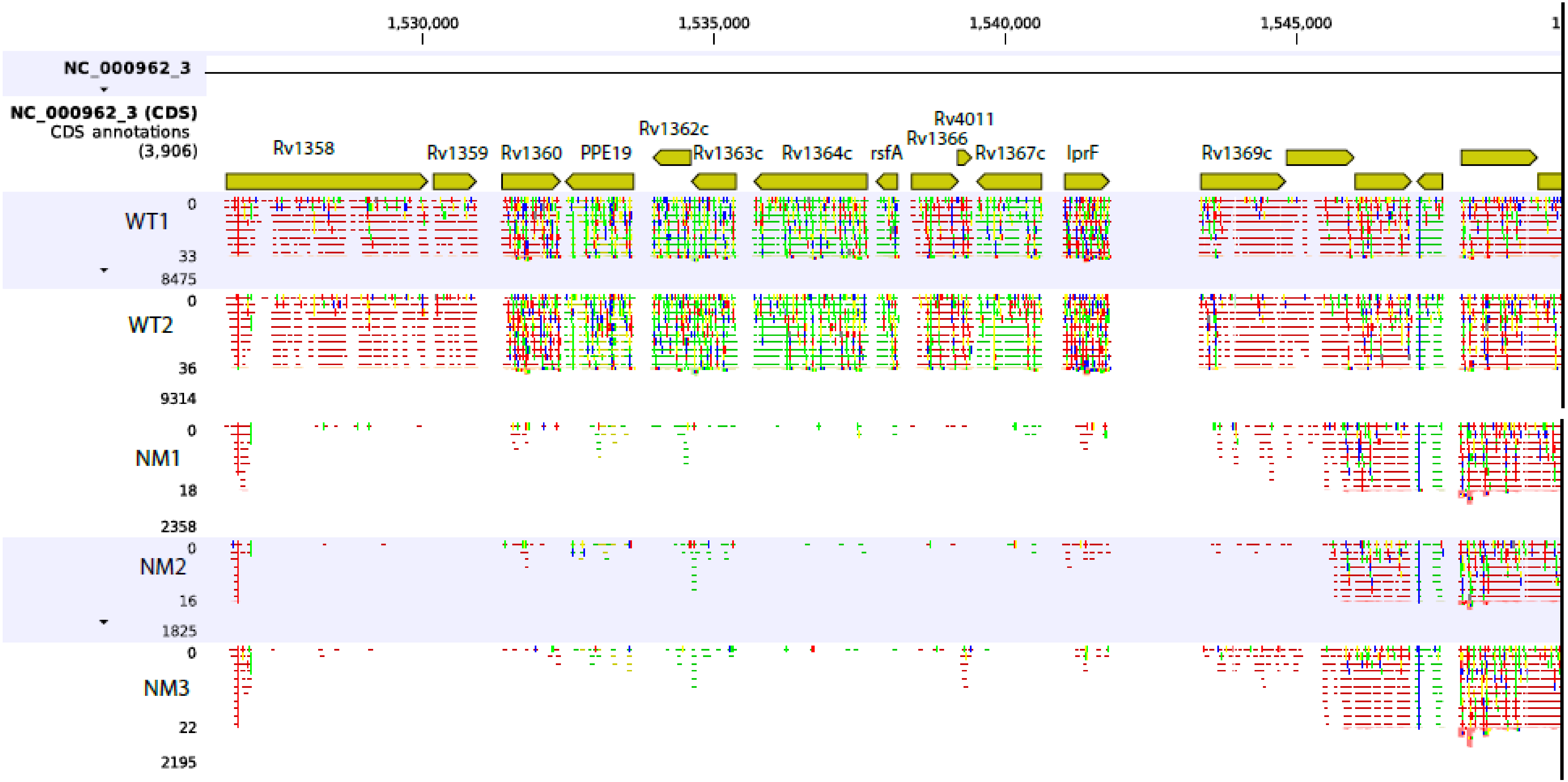
1,530,000
1,535,000
1,540,000
1,545,000
NC_000962_3
NC_000962_3 (CDS)
CDS annotations
(3,906)
Rv1358
Rv1359
Rv1360
PPE19
Rv1362c
Rv1363c
Rv1364c
rsfA
Rv4011
Rv1366
Rv1367c
lprF
Rv1369c
WT1
0
33
8475
WT2
0
36
9314
NM1
0
18
2358
NM2
0
16
1825
NM3
0
22
2195
