# Supplementary material for: Whole transcriptomic and proteomic analyses of an isogenic M. tuberculosis clinical strain with a naturally occurring 15 Kb genomic deletion
Source: PLoS One. 2017 Jun 26;12(6):e0179996. doi: 10.1371/journal.pone.0179996 (PMC5484546; doi:10.1371/journal.pone.0179996)
Supplement: S4 Fig — (PDF) [file pone.0179996.s008.pdf]

S4 Fig. STRING network of genes found down-regulated in the ON-A NM

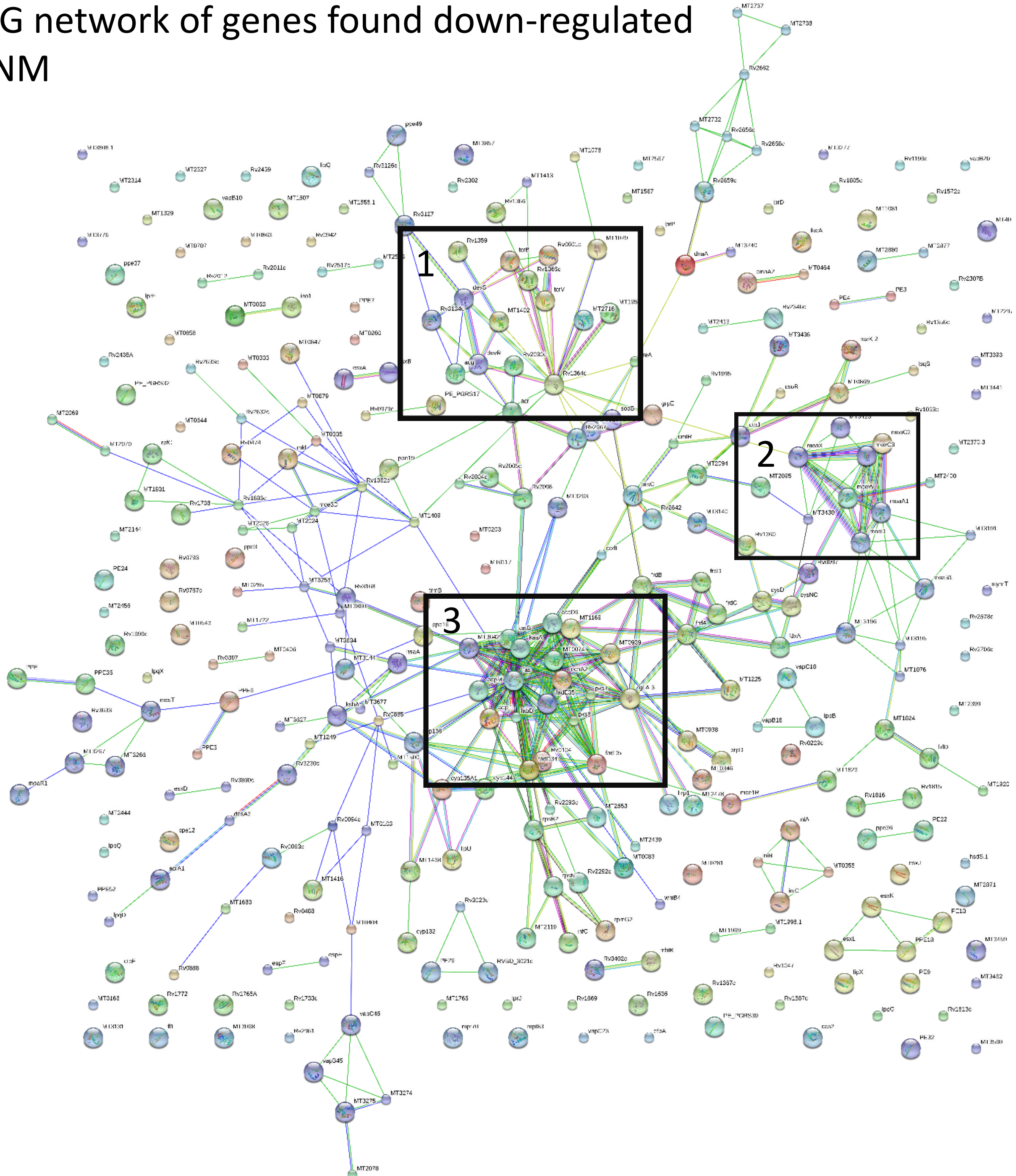

1. Regulatory genes

<u>DosR regulon:</u> *devR, devS, acg, acr, Rv2030c, Rv3127, Rv3134c, Rv3126.*

<u>Three component system:</u> *Rv0600 (tcrB), Rv0601c, Rv0602 (tcrV)*

<u>15Kb deletion</u>: *Rv1364c, rsfA*

2. Molybdenum Cofactor

moaX, moaC3, moaC2, moeWm moaA1, moaD, moaB1

3. Lipid Metabolism

<u>FAS-II pathway:</u> *kasA, kasB, accD6, acpM, fabD,*

<u>FAS-I:</u> *fas, acpA*

<u>Other lipid metabolism:</u> *fadD34, echA2, fadE35, pks7, pks8, MT1169 (Rv1135A), MT0939 (Rv0914c)*
