# Supplementary material for: Whole transcriptomic and proteomic analyses of an isogenic M. tuberculosis clinical strain with a naturally occurring 15 Kb genomic deletion
Source: PLoS One. 2017 Jun 26;12(6):e0179996. doi: 10.1371/journal.pone.0179996 (PMC5484546; doi:10.1371/journal.pone.0179996)
Supplement: S5 Fig — (PDF) [file pone.0179996.s009.pdf]

S5 Fig. STRING network of genes found up-regulated in the ON-A NM

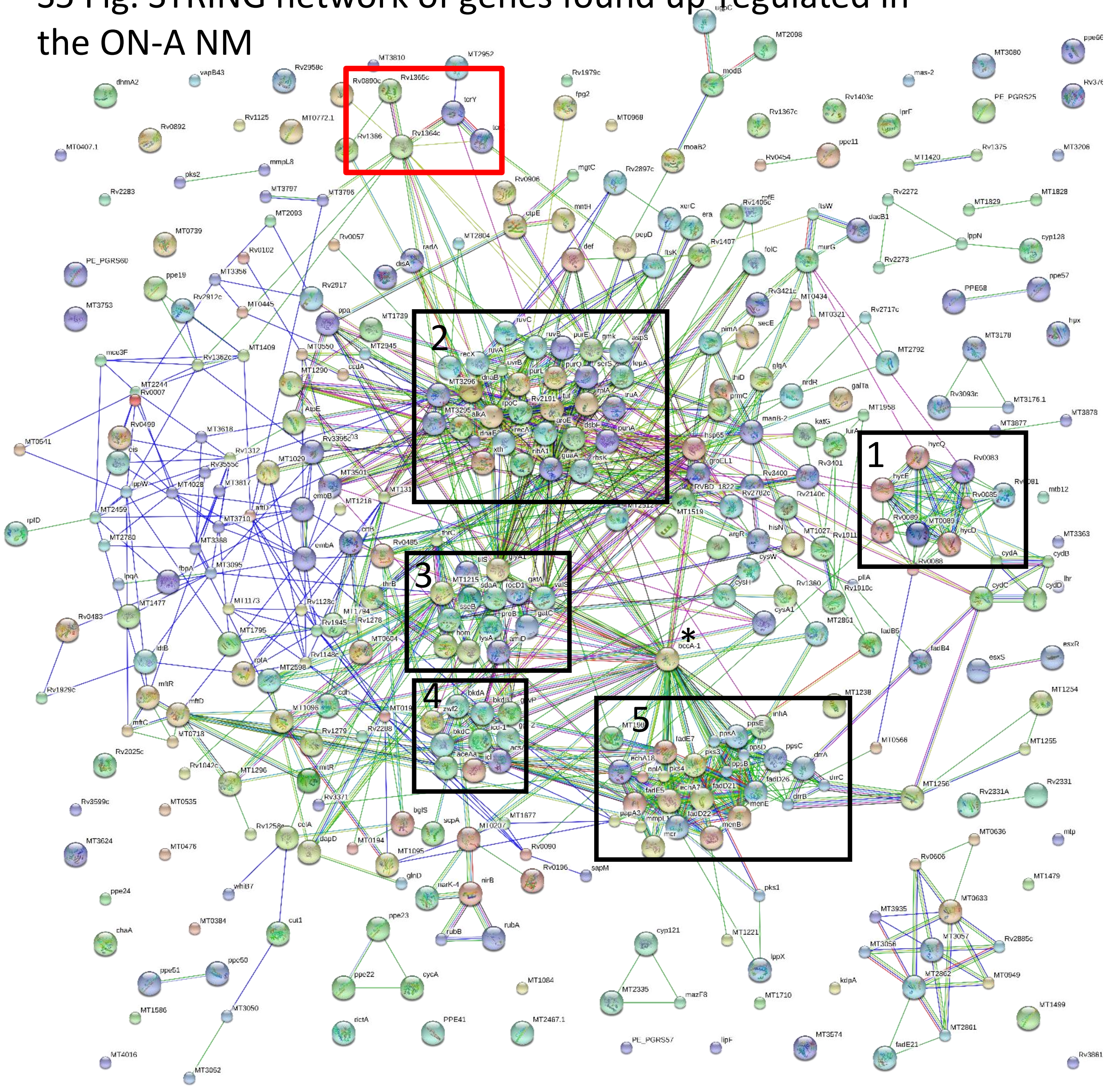

1. **Hyc operon**:
Rv0081, Rv0083, Rv0084 (hycD), hycP (Rv0085), Rv0086 (hycQ), Rv0087 (hycE), Rv0088, Rv0089

2. **Information Pathway:**
<u>DNA related:</u> *recA, recX, ruvABC, dnaE2, dnaB, uvrB, MT3296 (Rv3202c), MT3295 (Rv3201c)*
<u>Transcription:</u> *rpoC, Rv1407*
<u>Translation:</u> *rplA, lepA, era*
<u>t-RNA synthesis:</u> *valS, serS, aspS,*
Also includes enzymes involved in nucleotide synthesis: *purE, purQ, purL, gmk, guaA*

3. **Amino acid metabolism**:
lysA, hom, sdaA, thrBC, proB, gcvP, gatAC, glyA1, amiD, gatA, rocD, MT1215 (Rv1178)

4. ***Intermediate metabolism and respiration:***
bkdABC, acsA, aceAa, icl, icd-1, zwf2

5. ***Lipid metabolism:***
<u>PDIM synthesis and export:</u> *ppsABCDE, drrABC, lppX, fabD26*
<u>Menaquinone biosynthesis:</u> *menB, menE*
<u>Lipid degradation:</u> *fadD21, fadE7, fadE5, mcr*
<u>Other lipid synthesis:</u> *fadD22, echA7, echA18, inhA, papA3, pks4, pks1*
** bccA1 is a node for both, amino acid biosynthesis and lipid metabolism cluster.*

In red box, Rv1364-Rv1365 (RsfA)-Rv1366 (part of 15Kb deletion)
